# Biochemical Characterization of Uracil Phosphoribosyltransferase from *Mycobacterium tuberculosis*

**DOI:** 10.1371/journal.pone.0056445

**Published:** 2013-02-12

**Authors:** Anne Drumond Villela, Rodrigo Gay Ducati, Leonardo Astolfi Rosado, Carlos Junior Bloch, Maura Vianna Prates, Danieli Cristina Gonçalves, Carlos Henrique Inacio Ramos, Luiz Augusto Basso, Diogenes Santiago Santos

**Affiliations:** 1 Centro de Pesquisas em Biologia Molecular e Funcional (CPBMF), Instituto Nacional de Ciência e Tecnologia em Tuberculose (INCT-TB), Pontifícia Universidade Católica do Rio Grande do Sul (PUCRS), Porto Alegre, Rio Grande do Sul, Brazil; 2 Programa de Pós-Graduação em Biologia Celular e Molecular, Pontifícia Universidade Católica do Rio Grande do Sul (PUCRS), Porto Alegre, Rio Grande do Sul, Brazil; 3 Programa de Pós-Graduação em Medicina e Ciências da Saúde, Pontifícia Universidade Católica do Rio Grande do Sul (PUCRS), Porto Alegre, Rio Grande do Sul, Brazil; 4 Laboratório de Espectrometria de Massa, Empresa Brasileira de Pesquisa Agropecuária - Recursos Genéticos e Biotecnologia, Estação Parque Biológico, Brasília, Federal District, Brazil; 5 Instituto de Biologia, Universidade Estadual de Campinas (UNICAMP), Campinas, São Paulo, Brazil; 6 Instituto de Química, Universidade Estadual de Campinas (UNICAMP), Campinas, São Paulo, Brazil; Institute of Enzymology of the Hungarian Academy of Science, Hungary

## Abstract

Uracil phosphoribosyltransferase (UPRT) catalyzes the conversion of uracil and 5-phosphoribosyl-α-1-pyrophosphate (PRPP) to uridine 5′-monophosphate (UMP) and pyrophosphate (PP_i_). UPRT plays an important role in the pyrimidine salvage pathway since UMP is a common precursor of all pyrimidine nucleotides. Here we describe cloning, expression and purification to homogeneity of *upp*-encoded UPRT from *Mycobacterium tuberculosis* (*Mt*UPRT). Mass spectrometry and N-terminal amino acid sequencing unambiguously identified the homogeneous protein as *Mt*UPRT. Analytical ultracentrifugation showed that native *Mt*UPRT follows a monomer-tetramer association model. *Mt*UPRT is specific for uracil. GTP is not a modulator of *Mt*UPRT ativity. *Mt*UPRT was not significantly activated or inhibited by ATP, UTP, and CTP. Initial velocity and isothermal titration calorimetry studies suggest that catalysis follows a sequential ordered mechanism, in which PRPP binding is followed by uracil, and PP_i_ product is released first followed by UMP. The pH-rate profiles indicated that groups with p*K* values of 5.7 and 8.1 are important for catalysis, and a group with a p*K* value of 9.5 is involved in PRPP binding. The results here described provide a solid foundation on which to base *upp* gene knockout aiming at the development of strategies to prevent tuberculosis.

## Introduction

The major etiological agent of human tuberculosis (TB), *Mycobacterium tuberculosis*, currently infects one-third of the world's population. This pathogen was responsible for 8.5–9.2 million new TB cases in 2010, resulting in 1.5 million deaths worldwide [1]. Despite the availability of the Bacille Calmette-Guérin (BCG) vaccine and effective short-course chemotherapy, the increasing global burden of TB has been associated with co-infection with HIV [1], emergence of multi, extensively [2] and totally drug-resistant strains [3]. Furthermore, the ability of *M. tuberculosis* to remain viable within infected hosts in a long-term asymptomatic infection is an additional problem for the control of TB, since roughly 10% of people infected with latent TB develop the active form of the disease [4], [5]. There is thus a need for the development of new therapeutic strategies to control TB [6].

The complete genome sequencing of *M. tuberculosis* H37Rv has been an important progress towards a better understanding of the biology of bacilli and validation of molecular targets as candidates for rational drug design [7]. The knowledge of functional and structural features of enzymes involved in fundamental metabolic pathways is an important step for the target-based development of selective chemotherapeutic agents to treat TB [8]–[13]. Enzymes involved in pyrimidine biosynthesis have important roles in cellular metabolism, as they provide pyrimidine nucleosides that are essential components of a number of biomolecules [14]. Uridine 5′-monophosphate (UMP) is a common precursor of all pyrimidine nucleotides and can be synthesized either *de novo* from simple molecules or by the salvage pathway of preformed pyrimidine bases or nucleosides [15], [16]. Cells use the salvage pathway to reutilize pyrimidine bases and nucleosides because it represents a significant energy saving as the *de novo* synthesis is energy demanding [15], [16]. Uracil phosphoribosyltransferase (UPRT) is a key enzyme in the pyrimidine salvage pathway as it allows direct reutilization of uracil bases. Enzymes that catalyze the interconversion of uracil and uridine (uridine nucleosidase or uridine phosphorylase) and of uridine and UMP (uridine kinase or uridine monophosphatase) have not been identified by sequence homology in the *M. tuberculosis* genome [7]. Thus, UPRT appears to be the only operative enzyme that converts preformed pyrimidine bases to the nucleotide level [8].

**Figure 1:**
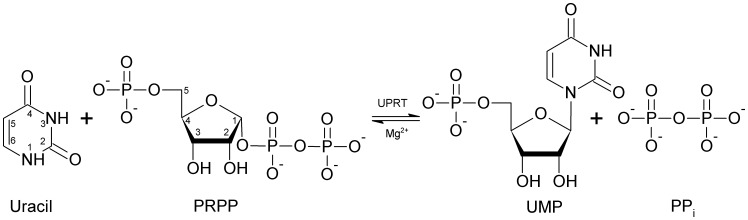
Chemical reaction catalyzed by *Mt*UPRT.

UPRTs catalyze the conversion of uracil and 5-phosphoribosyl-α-1-pyrophosphate (PRPP) to UMP and pyrophosphate (PP_i_) (Fig. 1). In *M. tuberculosis* H37Rv, two genes have been identified by sequence homology to likely encode proteins with UPRT activity (EC 2.4.2.9): *upp* (Rv3309c) and *pyrR* (Rv1379) [7]. Even though *pyrR* proteins are evolutionarily related to UPRTs as demonstrated by sequence and structural similarities, *M. tuberculosis pyrR* was shown to encode a protein with weak UPRT catalytic activity [17]. Thus, most of the UPRT activity and uracil salvage in *M. tuberculosis* probably arises from the *upp* gene product. Unlike enzymes from the *de novo* synthesis of UMP, UPRTs have mostly been characterized in lower organisms. Human UPRT has been isolated from the human fetal brain cDNA library [18]. However cloning, expression, and purification yielded a recombinant protein with no detectable UPRT catalytic activity [18]. Although there appears to be no solid experimental evidence for the presence of UPRT in humans, UMP can be obtained through uridine phosphorylase [19] reaction followed by uridine kinase activity, and uridine-cytidine kinase enzyme activity [20]. Human UMP synthase, a bifunctional protein containing both orotate phosphoribosyltransferase and orotidine-5′-phosphate decarboxylase activities [21], was shown to be preferentially used to activate 5-fluorouracil into its nucleotide [22]. Accordingly, the latter activity of this enzyme can also catalyze the conversion of uracil into UMP. Although the *upp* gene has been predicted to be non-essential by Himar 1-based transposon mutagenesis in the H37Rv strain [23], *M. tuberculosis* UPRT (*Mt*UPRT) might be an attractive target for the development of specific inhibitors due to its absence from the host. In addition, the apparently pivotal role of *Mt*UPRT in pyrimidine salvage pathway suggests that it may have a key role in the latent state and/or virulence of the tubercle bacilli. Thus, biochemical studies on *Mt*UPRT seem to be worth pursuing.

In this work, we present PCR amplification of *M. tuberculosis upp* gene, cloning, and purification to homogeneity of recombinant *Mt*UPRT. Mass spectrometry analysis and N-terminal amino acid sequencing confirmed the identity of recombinant *Mt*UPRT protein. Results on initial velocity measurements and isothermal titration calorimetry (ITC) data on substrate(s)/product(s) binding suggest that *Mt*UPRT follows a sequential ordered mechanism, in which PRPP binding is followed by uracil, and PP_i_ dissociation is followed by UMP release into solution. Analytical centrifugation results suggested a monomer-tetramer equilibrium process of *Mt*UPRT. pH-rate profiles provided the apparent p*K* values of amino acid residues involved in catalysis and substrate binding. The results described here may contribute to functional efforts towards a better understanding of *M. tuberculosis* biology, and provide a solid support on which to base gene replacement efforts.

## Results and Discussion

### PCR amplification and cloning of *M. tuberculosis upp* gene, and expression and purification of recombinant *Mt*UPRT

The 624 bp *upp* gene was amplified from *M. tuberculosis* H37Rv genomic DNA, cloned into the pCR-Blunt cloning vector, and subcloned into the pET-23a(+) expression vector between the *Nde*I and *Bam*HI restriction sites. Automatic DNA sequencing of the recombinant plasmid confirmed both identity and integrity of the *upp* gene, showing that no mutations were introduced during the PCR amplification steps.

**Figure 2:**
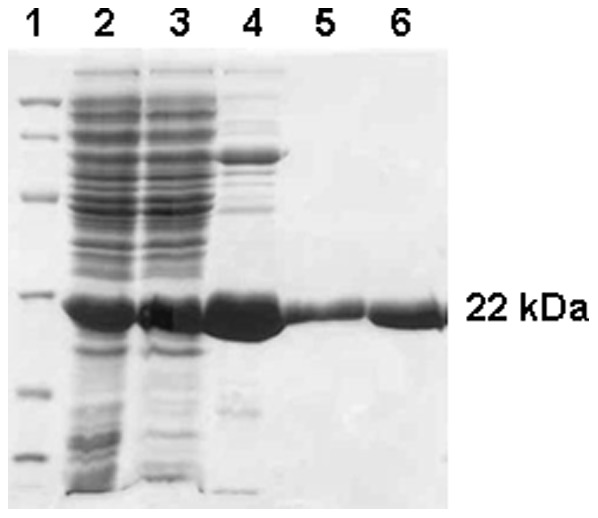
SDS-PAGE analysis of *Mt*UPRT purification steps. Lane 1, molecular weight protein marker; lane 2, crude extract; lane 3, sample loaded onto DEAE Sepharose CL6B column; lane 4, sample loaded onto Sephacryl S-200 column; lane 5, sample loaded onto Mono Q column; lane 6, homogeneous recombinant *Mt*UPRT eluted from the Mono Q column.

Sodium dodecyl sulfate-polyacrylamide gel electrophoresis (SDS-PAGE) analysis of BL21(DE3) *Escherichia coli* electrocompetent host cells transformed with recombinant pET-23a(+)::*upp* plasmid revealed that cell extracts contained a protein in the soluble fraction with an apparent molecular mass of 22 kDa (Fig. 2, lane 2). This is in agreement with the expected mass (21.898 kDa) of *Mt*UPRT (Expasy - compute pI/Mw programme kDa). Among the protocols tested, in our hands, the best experimental protocol for recombinant *Mt*UPRT protein expression was the following: BL21(DE3) *E. coli* host cells grown in Luria-Bertani (LB) medium at 37°C for 18 hours after cell culture reaching an OD_600 nm_ of 0.4 without isopropyl-β-D-thiogalactopyranoside (IPTG) induction. The pET expression vector system has a strong IPTG-inducible bacteriophage T7 *lacUV5* late promoter that controls the T7 RNA polymerase to transcribe cloned target genes [24]. However, *lac*-controlled systems could have high level protein expression in the absence of inducer due to derepression of the system when cells approach stationary phase in complex medium, as previously reported for other enzymes [19], [25]–[30].

*Mt*UPRT was purified to homogeneity by three steps of liquid chromatography. The purification protocol included an anion-exchange column (DEAE Sepharose CL6B), a gel filtration column (Sephacryl S-300), followed by desorption of homogeneous *Mt*UPRT protein from a strong anion-exchange column (Mono Q) as assessed by SDS-PAGE (Fig. 2). This 2.1-fold purification protocol yielded 20 mg of homogeneous *Mt*UPRT from 2 g of wet cells, indicating a 31% protein yield (Table 1). Enzyme activity assays confirmed that recombinant *Mt*UPRT catalyses the conversion of uracil and PRPP to UMP and PP_i_. Homogeneous *Mt*UPRT was stored at −80°C with no loss of activity for up to 1 year.

**Table 1:** Purification of *Mt*UPRT from *E. coli* BL21(DE3) electrocompetent host cells.^a^

Purification step	Total protein (mg)	Total enzyme activity (U)	Specific activity (U mg^−1^)	Purification fold	Yield (%)
Crude extract	132.74	100.47	0.76	1.0	100
DEAE Sepharose CL6B	86.70	95.92	1.11	1.5	95
Sephacryl S-300	41.32	35.63	0.86	1.1	35
Mono Q	20.23	31.61	1.56	2.1	31

a Typical purification protocol starting from 2 g of wet cells.

### Mass spectrometry analysis and N-terminal amino acid sequencing

The *Mt*UPRT subunit molecular mass was determined by mass spectrometry analysis to be 21,898.1 Da, consistent with the expected molecular mass of 21,898.2 Da (Expasy - compute pI/Mw programme). The predicted subunit molecular mass of *E. coli* UPRT is 23,500 Da. The first 51 N-terminal *Mt*UPRT amino acid residues identified by the Edman degradation method correspond to those predicted for the *upp* gene protein product. These results unambiguously identify the homogeneous recombinant protein as *Mt*UPRT.

### Determination of oligomeric state of *Mt*UPRT in solution

**Figure 3:**
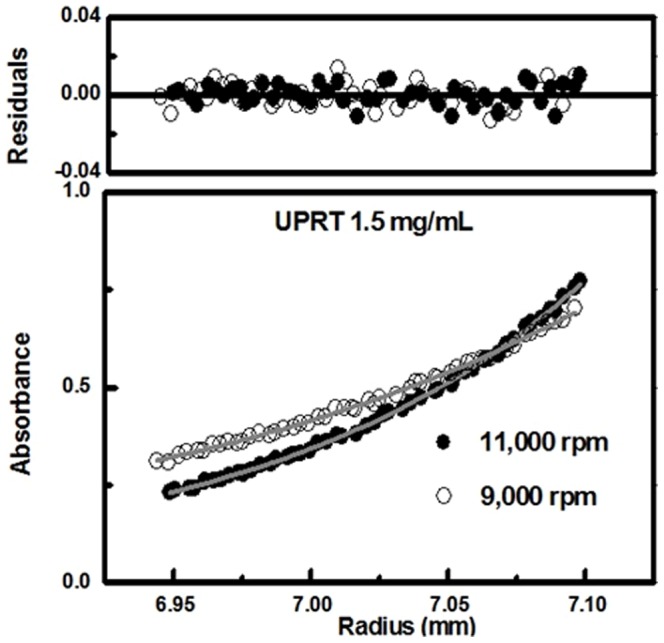
Sedimentation equilibrium experiment. A model (equation) of absorbance versus cell radius was fitted to the data by applying nonlinear regression. The experimental data for 1.5 mg/mL of protein at 9,000 and 11,000 rpm are shown. The random distribution of the residues (top panel) indicated a good quality fit in agreement with monomer-tetramer equilibrium.

The molecular mass of native *Mt*UPRT was determined by the sedimentation equilibrium (SE) method of analytical ultracentrifugation (AUC). The molecular mass of a sedimenting particle was derived independently of sedimentation and diffusion coefficients and obtained from fitting the concentration distribution of macromolecules at equilibrium. The experiment was carried out with protein concentrations ranging from 0.5 to 1.5 mg mL^−1^ and rotor speed from 3,000 to 11,000 rpm at 4°C with scan data acquisition at 275 nm. A model (equation) of absorbance versus cell radius was fitted to the data by applying nonlinear regression using Origin software. The best results were obtained with 1.5 mg mL^−1^ of protein at 9,000 and 11,000 rpm which were determined by the distribution randomness of residuals and by the minimization of variance (3.8×10^−5^). Variances for single species were: monomer, 1.8×10^−4^; dimer, 9.2×10^−5^; trimer, 4.7×10^−5^; and tetramer, 4.1×10^−5^. The random distribution of residuals (Fig. 3) indicates appropriate fitting and is in agreement with the monomer-tetramer association model, with an estimated equilibrium dissociation constant of approximately 10^−6^ M.

**Figure 4:**
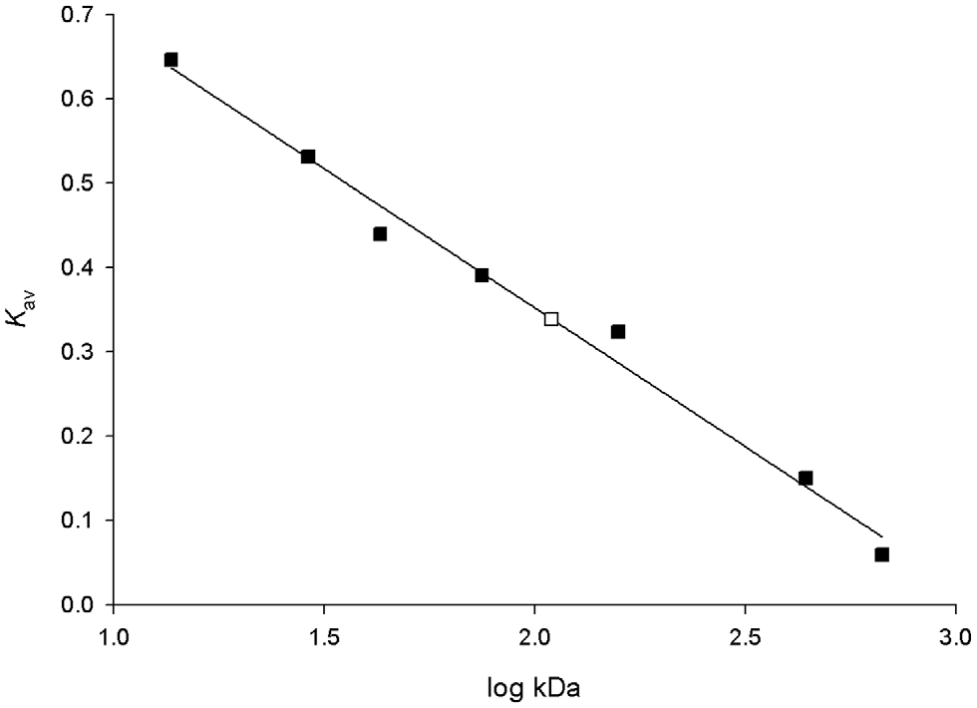
Calibration curve of Superdex 200 HR column with protein standards. The following standards were employed (solid squares): ribonuclease A (13,700 Da), carbonic anhydrase (29,000 Da), ovalbumin (43,000 Da), conalbumin (75,000 Da), aldolase (158,000 Da), ferritin (440,000 Da) and thyroglobulin (669,000 Da). The *K*_av_ value was calculated for each standard protein using the equation (*V*_e_ – *V*_0_)/(*V*_t_ – *V*_0_), where is *V*_e_ the elution volume for the protein and *V*_t_ is the total bed volume, and *K*_av_ was plotted against the logarithm of standard molecular weights. The experimental *K*_av_ (open square) suggests a value of 109,650 Da for the molecular mass of recombinant *Mt*UPRT in solution.

A value of 109,650 Da for the molecular mass of homogeneous *Mt*UPRT protein was estimated by size exclusion chromatography (Fig. 4). This result suggests that *Mt*UPRT is a pentamer or tetramer in solution, in agreement with AUC results. Even though *Mt*UPRT was shown to be present in a monomer-tetramer equilibrium model by AUC, the tetramer seems to be more abundant as no monomer could be detected by size exclusion chromatography.

Different oligomeric states were found for UPRTs from several organisms. The *Toxoplasma gondii* UPRT behaved as a dimer in solution, whereas in the presence of guanosine 5′-triphosphate (GTP), the enzyme is a tetramer [31]. *Sulfolobus solfataricus* and *Sulfolobus shibatae* UPRTs present tetrameric oligomeric states [32], [33], whereas both *Giardia intestinalis* [34] and *Bacillus caldolyticus* [35] enzymes are dimeric proteins. *E. coli* UPRT was shown to be a dimer or trimer in the absence of ligands, while in the presence of PRPP and GTP it was shown to be a pentamer or hexamer with both forms existing in a dynamic equilibrium [36], [37].

### Substrate specificity, apparent steady-state kinetic parameters, and evaluation of nucleotides as allosteric effectors

Prior to embarking on determination of the true steady-state kinetic parameters and *Mt*UPRT enzyme mechanism, studies on substrate specificity, assessment of apparent steady-state kinetic parameters, and evaluation of nucleotides as possible allosteric effectors were carried out.

#### Evaluation of pyrimidine bases as substrates

Uracil, thymine and cytosine pyrimidine bases were evaluated as possible *Mt*UPRT substrates. The bases were added to *Mt*UPRT reaction mixtures and protein separated by ultrafiltration, and product formation analyzed by HPLC monitoring absorbance at 254, 260, and 280 nm. The results show that *Mt*UPRT is specific for uracil, as no product formation could be detected for both cytosine and thymine bases (data not shown). This result was confirmed using liquid chromatography coupled to electrospray ionization tandem mass spectrometry (LC-ESI-MS/MS) [38]. UPRT from several organisms were also shown to be specific for uracil and some uracil analogues [33], [36]. The *Mt*UPRT enzyme activity measurements henceforth described were carried out using uracil as substrate and a continuous spectrophotometric assay.

#### Apparent steady-state kinetic parameters

**Figure 5:**
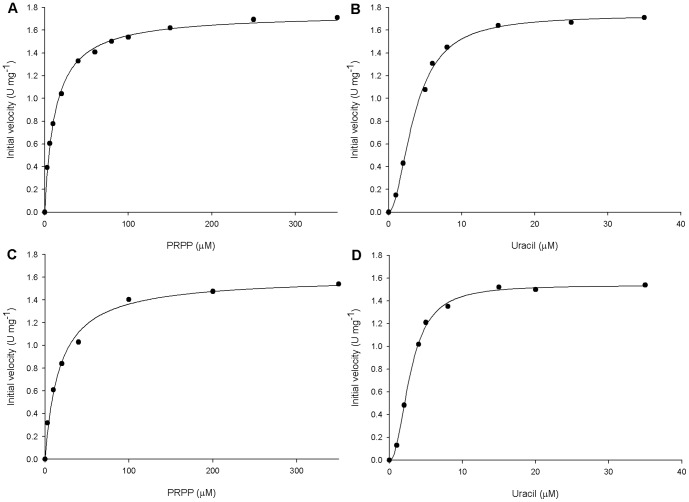
Apparent steady-state kinetic parameters. (A) Initial velocity of *Mt*UPRT (U mg^−1^) as a function of increasing PRPP concentration in the presence of constant uracil concentration (10 µM). (B) Initial velocity of *Mt*UPRT as a function of increasing uracil concentration in the presence of constant PRPP concentration (100 µM). (C) Initial velocity of *Mt*UPRT as a function of increasing PRPP concentration in the presence of constant concentrations of uracil (10 µM) and GTP (100 µM). (D) Initial velocity of *Mt*UPRT as a function of increasing uracil concentration in the presence of constant concentrations of PRPP (100 µM) and GTP (100 µM).

The dependence of initial velocity on PRPP as a variable substrate at fixed-saturating uracil concentration (35 µM) followed hyperbolic Michaelis-Menten kinetics [39] (Fig. 5A). Accordingly, the data were fitted to the Michaelis-Menten equation 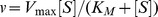, in which *v* is the steady-state velocity, *V*_max_ is the maximal rate, [S] is the substrate concentration, and *K*_M_ is the Michaelis-Menten constant. This analysis yielded the following values for the apparent constants: *K*_M_ = 12.4±0.6 µM and *V*_max_ = 1.75±0.02 U mg^−1^ (*k*_cat_ = 0.65±0.01 s^−1^). The saturation curve for uracil at a fixed-saturating PRPP concentration (350 µM) was sigmoidal (Fig. 5B). These data were thus fitted to the Hill equation 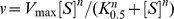, in which *v* is the measured reaction velocity, *V*_max_ is the maximal velocity, *S* is the substrate concentration, *n* is the Hill coefficient (indicating the cooperative index), and *K*_0.5_ is the substrate concentration in which *v* = 0.5*V*_max_. Data fitting to the Hill equation yielded the following values for uracil: *K*_0.5_ = 3.6±0.1 µM, *V*_max_ = 1.73±0.03 U mg^−1^ (*k*_cat_ = 0.64±0.01 s^−1^), and *n* = 1.9±0.1. The positive value for *n* indicates positive homotropic cooperativity for uracil. Although *B. caldolyticus* UPRT displayed hyperbolic saturation curve [35], the *K*_M_ value for uracil (2 µM) is similar to the *K*_0.5_ here reported.

Apparent steady-state kinetic parameters were also determined in the presence of 100 µM GTP (Fig. 5C and D). The values were *K*_M = _17±2 µM and *V*_max_ = 1.60±0.04 U mg^−1^ (*k*_cat_ = 0.59±0.01 s^−1^) for hyperbolic saturation curve for PRPP as the variable substrate at fixed-saturating uracil concentration (35 µM); and *K*_0.5 = _2.9±0.1 µM, *V*_max_ = 1.54±0.02 U mg^−1^ (*k*_cat_ = 0.57±0.01 s^−1^), and *n* = 2.2±0.1 for sigmoidal saturation curve for uracil as the variable substrate at fixed-saturating PRPP concentration (350 µM).

**Figure 6:**
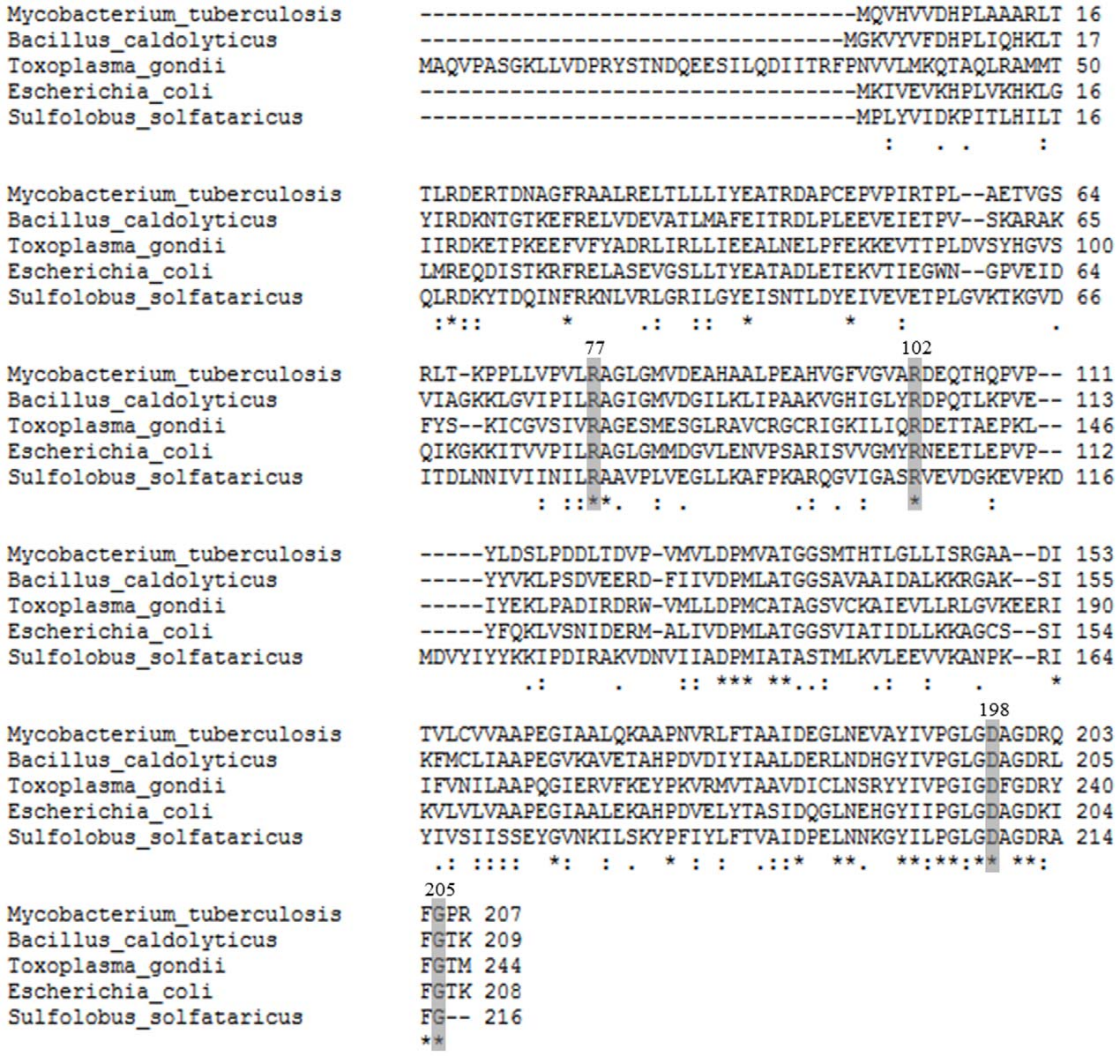
Multiple sequence alignment of amino acid sequences of UPRTs from *M. tuberculosis*, *B. caldolyticus*, *T. gondii*, *E. coli* and *S. solfataricus*. Amino acids for each polypeptide sequence were independently numbered. Identical conserved residues are indicated by stars below the alignment. Residues proposed to be involved in catalysis (ARg102 and Asp198), PRPP substrate binding (ARg77 and Arg102), and (or not) C-terminal glycine (Gly205) are highlighted (*Mt*UPRT numbering). Multiple sequence alignment was carried out using Clustal W2 software (http://www.ebi.ac.uk/Tools/msa/clustalw2/).

In contrast to reports on UPRTs from *E. coli* [36], [37], *S. solfataricus* [32] and *S. shibatae* [33], the kinetic parameters for *Mt*UPRT were not affected by GTP (Fig. 5C and D). GTP lowered the *K*_M_ value for PRPP, changing saturation curves from slightly sigmoidal to strictly hyperbolic without affecting *V*_max_ for *E. coli* UPRT [37]. GTP was also shown to cause a dramatic increase in the activity of *G. intestinalis* UPRT [34]. The enzyme from *T. gondii* was shown to be activated by GTP, which also stabilizes the more active tetrameric form of the enzyme [31]. GTP was shown to increase *k*_cat_ and *K*_M_ values for PRPP and uracil of *S. solfataricus* UPRT, whereas cytidine 5′-triphosphate (CTP) inhibited the enzyme in the presence of UMP [32]. UPRTs whose enzyme activities are regulated by GTP and CTP are truncated with a conserved C-terminal glycine residue [40]. It has been shown that extending the polypeptide chain from the C-terminal glycine by adding a threonine and methionine to *S. solfataricus* UPRT resulted in an endogenously activated mutant protein since high activity was detected in the absence of GTP [40]. This result is in agreement with UPRT enzymes from other organisms whose activity are not regulated by GTP, and that have the conserved C-terminal glycine residue followed by one or a few more amino acid residues [40]. *B. caldolyticus* UPRT and *Mt*UPRT, which possess an amino acid sequence identity of approximately 45%, have 2 amino acid residues after the conserved glycine (Fig. 6), which might be the reason for GTP not having any effect on the activity of these enzymes. However it should be pointed out that GTP activates UPRTs from distinct organisms in a different manner. An example is the UPRT from *T. gondii* which possesses additional residues (Fig. 6) and is still GTP activated by influencing the oligomeric state [31], in contrast to *S. solfataricus* enzyme, which GTP binding affects *k*_cat_ and *K*_M_ values of the tetrameric enzyme [40].

#### Evaluation of nucleotides as allosteric effectors

**Figure 7:**
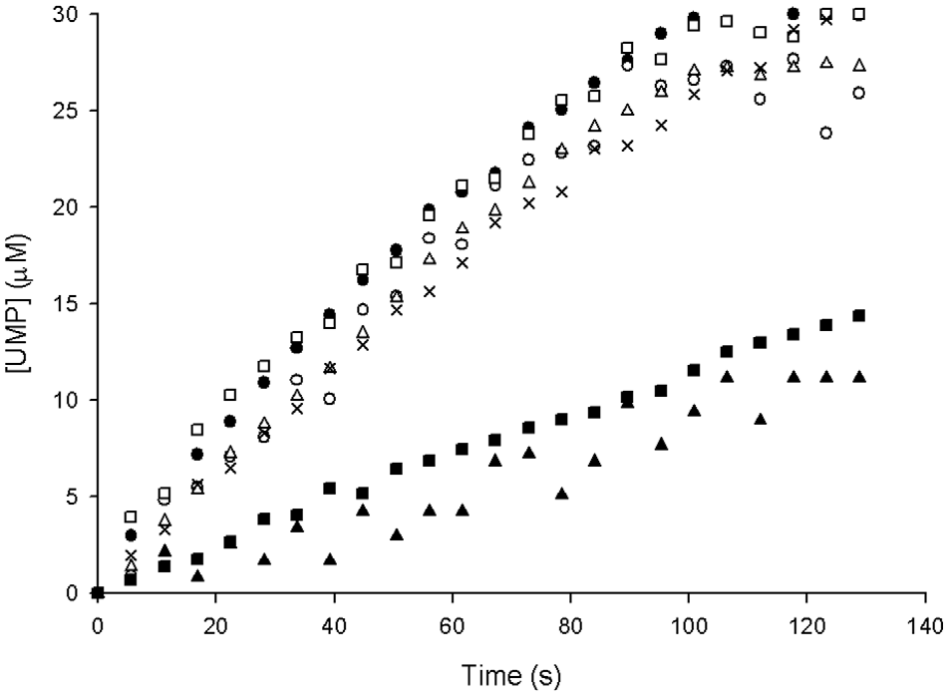
Evaluation of nucleotides as allosteric effectors. All reactions contained 350 µM PRPP and 35 µM uracil. (•) standard reaction, (○) standard reaction containing 500 µM GTP, (□) standard reaction containing 500 µM CTP, (Δ) standard reaction containing 500 µM ATP, (×) standard reaction containing 500 µM UTP, (▪) standard reaction containing 100 µM UMP, (▴) standard reaction containing both 100 µM UMP and 500 µM CTP.

UPRTs from several organisms are allosterically regulated by nucleotides. Accordingly, a number of nucleotides were evaluated as possible allosteric effectors of *Mt*UPRT by monitoring the enzyme-catalyzed chemical reaction for 128 s (Fig. 7). The absorbance was converted to UMP concentration using the following equation: *C* = *A*/Δε *b*, where *C* is the UMP concentration, *A* is the absorbance at 280 nm, Δε is the molar absorptivity based on differential absorption between uracil and UMP (Δε = 2.5×10^3^ M^−1^ cm^−1^), and *b* is the optical path (0.5 cm). *Mt*UPRT was not significantly activated or inhibited by 500 µM of any of the following nucleotides: adenosine 5′-triphosphate (ATP), uridine 5′-triphosphate (UTP), GTP, and CTP. As expected, enzyme inhibition was observed in the presence of 100 µM of the product UMP. However, no increase in UMP inhibition occurred in the presence of CTP, as has been reported for *S. solfataricus* UPRT [32].

### Determination of *Mt*UPRT kinetic mechanism

Initial velocity patterns and isothermal titration calorimetry (ITC) of ligand binding to *Mt*UPRT were employed to assess the enzyme mechanism.

#### Initial velocity pattern

**Figure 8:**
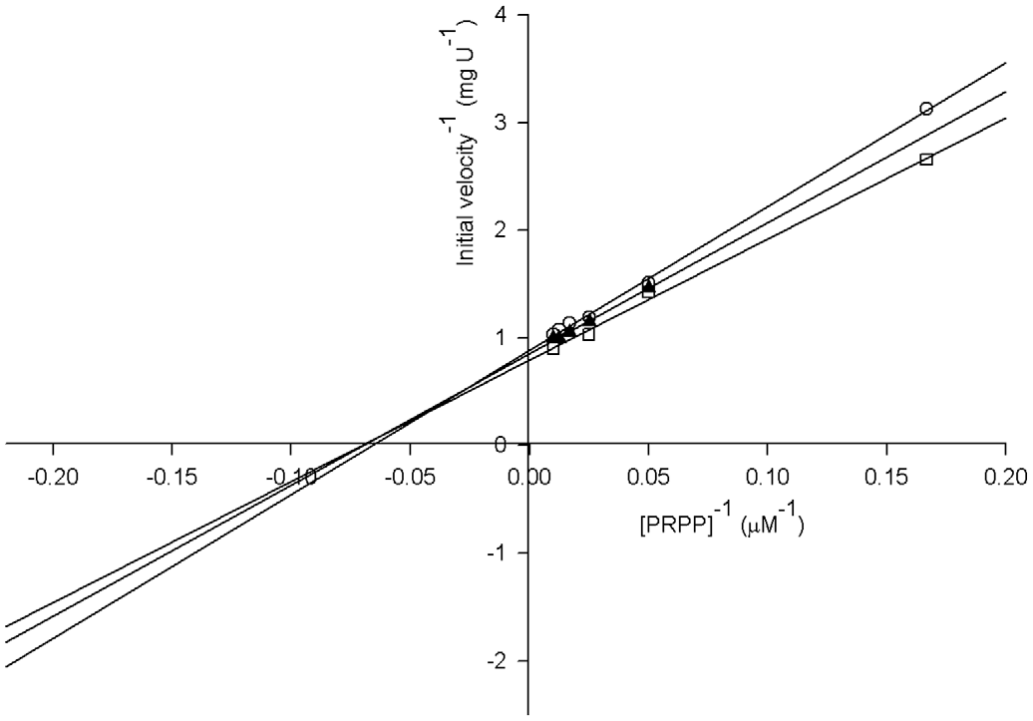
Initial velocity patterns for *Mt*UPRT. Double-reciprocal plot of enzyme initial velocity^−1^ (mg U^−1^) versus [PRPP]^−1^ (µM^−1^). Concentrations of uracil were: 6 µM (open circles), 8 µM (filled triangle), and 10 µM (open squares).

The initial velocity pattern for the *Mt*UPRT catalyzed reaction at varying concentrations of PRPP at fixed-varying uracil concentrations is shown in Fig. 8, as a double-reciprocal plot (Lineweaver-Burk plot). A pattern of intersecting lines to the left of *y*-axis (Fig. 8) was observed for PRPP, which is consistent with ternary complex formation and a sequential mechanism [41]. The plots of *Mt*UPRT activity versus uracil concentration in the presence of different PRPP concentrations were all sigmoidal (data not shown), thereby giving non-linear double-reciprocal plots that precluded the analysis based on patterns of lines. Accordingly, the only enzyme mechanism that could be ruled out is the ping-pong (double-displacement) that gives a parallel pattern of lines. At any rate, the pattern of intersecting line given in Fig. 8 indicates that productive catalysis only occurs when both substrates are bound to the enzyme active site [42]. The data (Fig. 8) were fitted to *v* = *VAB*/(*K*_ia_ *KB*+*K*_a_ *B*+*K*_b_ *A*+*AB*), yielding the following true steady-state kinetic parameters: *k*_cat_ = 0.58±0.02 s^−1^, *K*_PRPP_ = 14±1 µM, *K*_uracil = _2.6±0.4 µM, *k*_cat_/*K*_PRPP_ = 4.1 (±0.2)×10^4^ M^−1^ s^−1^, and *k*_cat_/*K*_uracil_ = 2.2 (±0.3)×10^5^ M^−1^ s^−1^. The initial velocity pattern obtained (Fig. 8) could not be improved due to experimental limitations.

#### Equilibrium binding of ligands to *Mt*UPRT assessed by ITC

**Figure 9:**
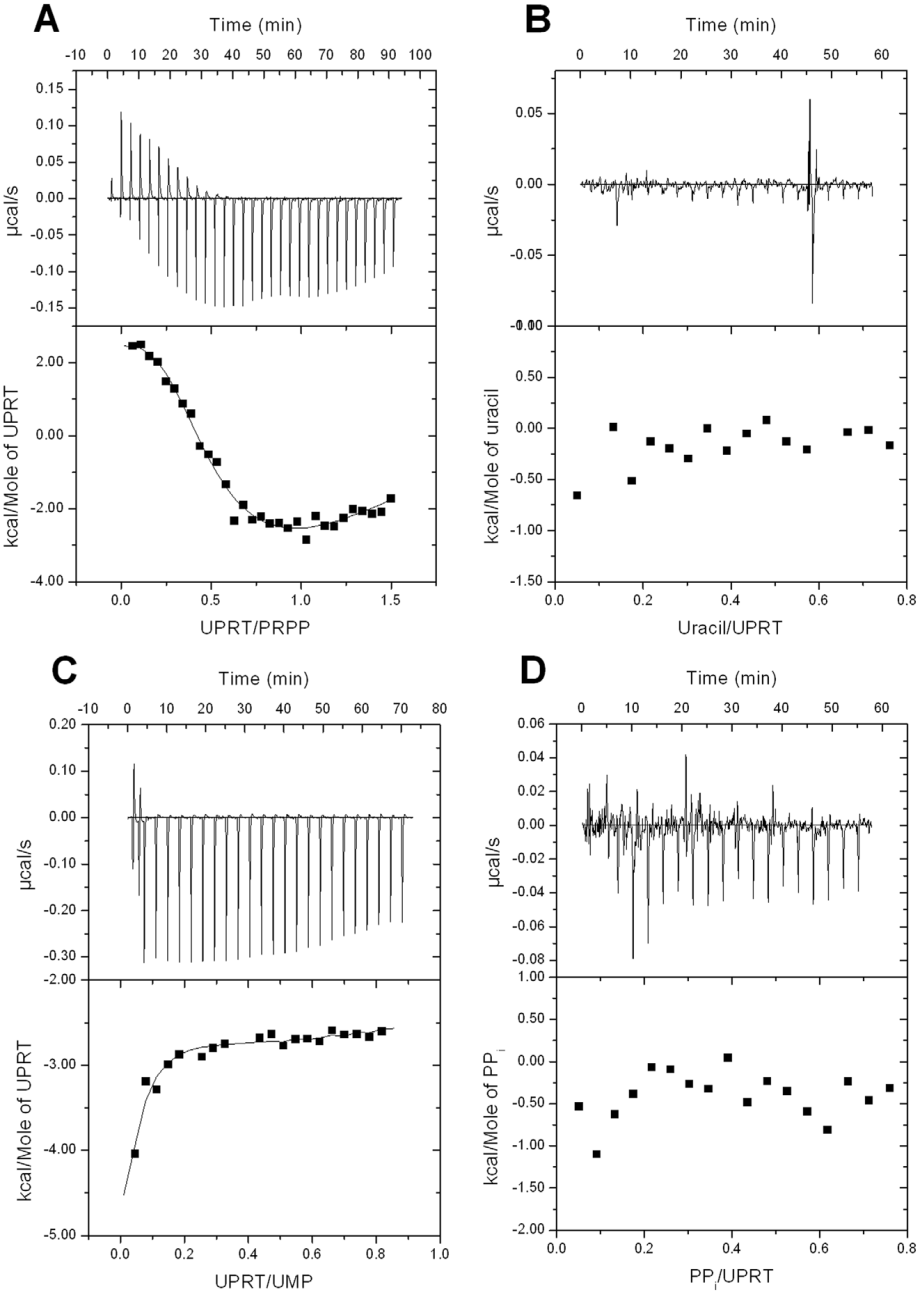
Isothermal titration (ITC) curves of binding of ligands to *Mt*UPRT. (A) Reverse titration of PRPP substrate. (B) Titration of uracil substrate. (C) Reverse titration of UMP product. (D) Titration of PP_i_ product.

To try to ascertain whether or not there is an order of substrate addition to *Mt*UPRT, ITC experiments were carried out. ITC was also employed to evaluate the relative affinity of ligand binding to free *Mt*UPRT enzyme. ITC measures the heat that is transferred upon formation of a ligand-macromolecule complex at a constant temperature and pressure. The measure of the heat released/or taken up upon binding of the ligand allows determination of the association constant (*K*_a_) and the binding enthalpy (Δ*H*) of the process. The dissociation constant at equilibrium (*K*_d_) is calculated as the inverse of *K*_a_ (*K*_d_ = 1/*K*_a_). Moreover, the entropy of the binding reaction (Δ*S*) and the Gibbs free energy (Δ*G*) are obtained from the equation: Δ*G* = −*RT*ln*K*_a_ = Δ*H*–*T*Δ*S*, where *R* is the gas constant (8.314 J K^−1^ mol^−1^) and *T* is the temperature in Kelvin (*T* = °C+273.15) [43]. The heat change upon binding for each individual injection was plotted as a function of the molar ligand-to-protein ratio. To derive the thermodynamic parameters, the equation for four-site sequential binding model was used to fit the data as it provided the best fit for ITC results. The four-site model is also consistent with the native molecular mass of *Mt*UPRT determined by AUC and size exclusion chromatography. PRPP (Fig. 9A) and UMP (Fig. 9C) binding isotherms to free *Mt*UPRT showed significant heat changes, providing a thermodynamic signature of non-covalent interactions for each ligand and allowing the determination of the thermodynamic parameters for each binding site (Table 2). Direct and reverse titrations with PRPP and UMP were conducted to check the stoichiometry and the suitability of the model [44]. Since the direct titrations generated large standard errors for the thermodynamic parameters, only the reverse titrations for PRPP (Fig. 9A) and UMP (Fig. 9C) are presented here. The binding of PRPP to free *Mt*UPRT enzyme generated both exothermic and endothermic profiles, exhibiting a biphasic behavior (Fig. 9A), while the binding of UMP exhibited an exothermic binding process (Fig. 9C). Notwithstanding, the affinity of binding for both PRPP and UMP among *Mt*UPRT subunits were similar, except subunit 2 bound to PRPP (Table 2). The thermodynamic analysis revealed different types of interactions between the ligand and enzyme subunits. Negative enthalpy suggests favorable hydrogen bond contacts or van der Waals interactions. Negative entropy implies conformational changes, whereas positive entropy indicates that the reaction is dominated by solvent rearrangement and hydrophobic forces [43]. The signature of non-covalent interactions leading to *Mt*UPRT:PRPP binary formation suggests that the first and second binding processes are guided by the release of “bound” water molecules. The third and fourth binding processes suggest that there may be favorable hydrogen bond formation or van der Waals interactions (negative Δ*H*), followed by an unfavorable redistribution of the hydrogen bond network between the reacting species (positive Δ*H*). In addition, the third process of PRPP binding appears to be associated with conformational changes in either the ligand or protein (negative Δ*S*), and the fourth process appears to be dominated by the release of water molecules to the bulk solvent (positive Δ*S*) [43]. The non-covalent signatures of *Mt*UPRT:UMP complex formation processes are somewhat similar to PRPP. At any rate, the Δ*G* values are similar and all binding processes are favorable (negative Δ*G*) for PRPP and UMP.

**Table 2:** Thermodynamic parameters of PRPP and UMP ligands binding to *Mt*UPRT.^a^

Ligands	*K*_a_ (M^−1^)	Δ*H* (kcal mol^−1^)	Δ*S* (cal mol^−1^ deg^−1^)	Δ*G* (kcal mol^−1^)	*K*_d_ (µM)
PRPP					
Subunit 1	2.0 (±3.6)×10^5^	2.8±0.3	34±6	−7±1	5.0±0.9
Subunit 2	2.1 (±0.6)×10^4^	−3±16	10±3	−6±2	48±15
Subunit 3	1.6 (±0.5)×10^5^	−41±32	−114±33	−7±2	6±2
Subunit 4	1.1 (±0.4)×10^5^	58±22	217±85	−7±3	9±4
UMP					
Subunit 1	7 (±2)×10^4^	−5.1±0.4	5±1	−7±2	14±3
Subunit 2	1.5 (±5)×10^5^	6±3	45±16	−7±2	7±2
Subunit 3	1.2 (±0.4)×10^5^	−24±11	−56±21	−7±3	8±3
Subunit 4	8 (±3)×10^4^	28±18	117±43	−7±2	13±5

a *K*_a = _association constant; Δ*H = *binding enthalpy; Δ*S = *binding entropy; Δ*G = *Gibbs free energy; *K*_d_ = dissociation constant.

The ligand binding isotherms showed no significant heat changes upon either uracil (Fig. 9B) or PP_i_ (Fig. 9D) interaction with free *Mt*UPRT enzyme. These data suggest that both uracil and PP_i_ cannot bind to free enzyme. Furthermore, no binding of GTP either to the free enzyme or to the PRPP bound enzyme was detected by ITC (data not shown). The incubation of PRPP with *Mt*UPRT prior to the titration of GTP was tested to determine whether the binding of PRPP to *Mt*UPRT generates conformational changes on the enzyme that could enable GTP binding. However, no binding of GTP to *Mt*UPRT:PRPP binary complex could be detected (data now shown). These results are in agreement with the steady-state kinetic results showing that GTP has no effect on *Mt*UPRT enzyme activity, and therefore *Mt*UPRT is not allosterically regulated by this nucleotide.

#### Proposed kinetic mechanism

**Figure 10:**
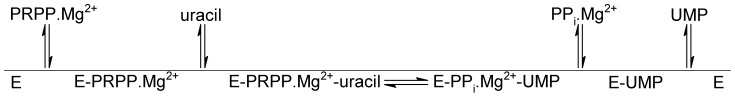
Proposed kinetic mechanism for *Mt*UPRT. This order of substrate binding and product release is suggested on the basis of thermodynamic results.

The initial velocity pattern of intersecting lines (Fig. 8) suggested a sequential mechanism (either random or ordered). On the other hand, the ITC data allowed determination of order of substrate addition and product release (Fig. 10). Accordingly, the *Mt*UPRT enzyme mechanism consistent with these results is ordered addition of substrate, in which binding of PRPP precedes the binding of uracil, and ordered product release, PP_i_ release from *Mt*UPRT:UMP:PP_i_ ternary complex is followed by UMP release to yield free enzyme for the next round of catalysis (Fig. 10). It should be pointed out that this order of substrate binding and product release is suggested on the basis of thermodynamic and not kinetic results. Ordered sequential mechanisms of substrate binding have been reported for *E. coli* [45], *S. solfataricus* [32], *G. intestinalis* [34] and *B. caldolyticus* [16] UPRTs.

### pH-rate profiles

**Figure 11:**
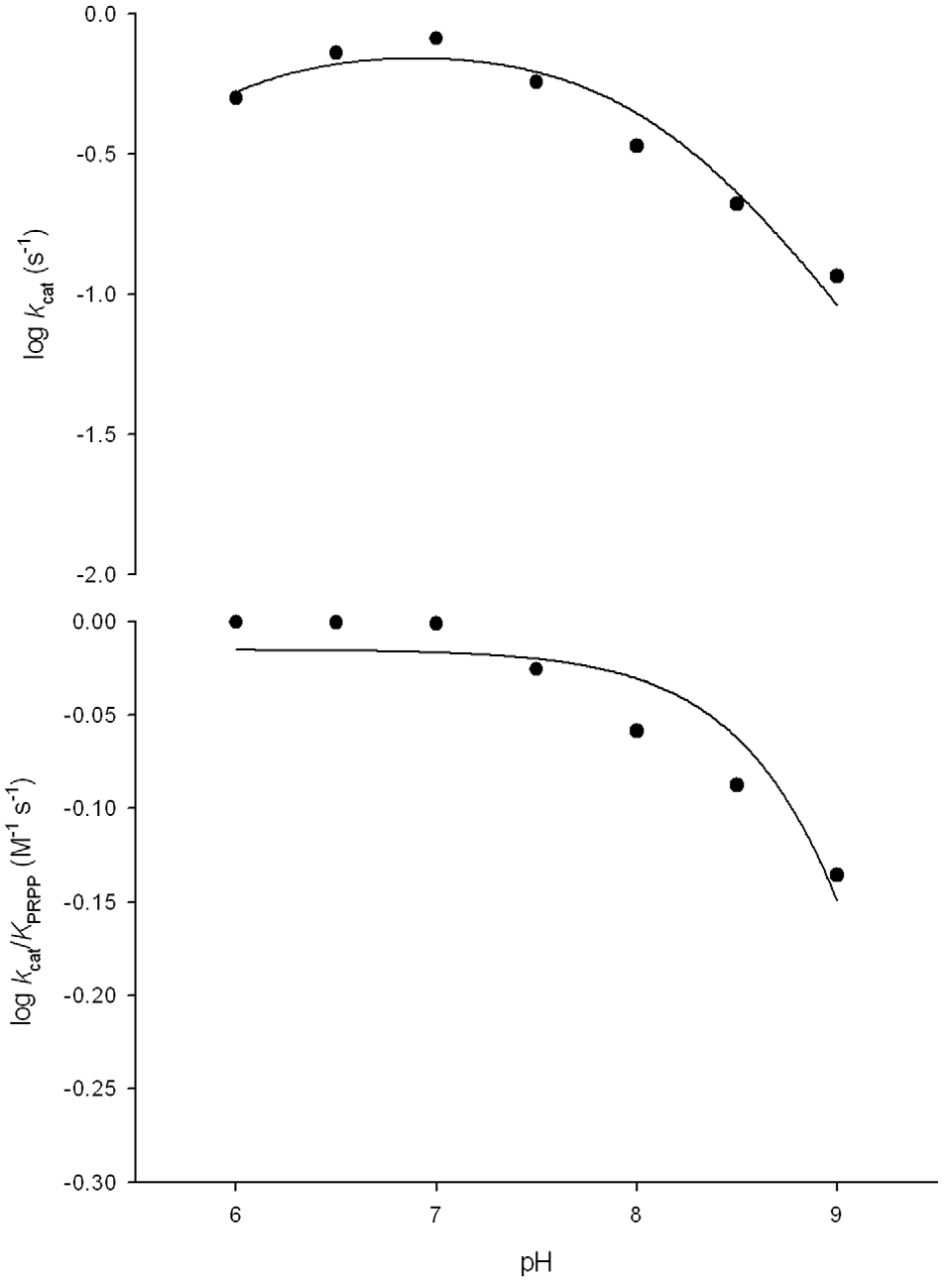
Dependence of kinetic parameters on pH. (A) pH dependence of log *k*_cat_. (B) pH dependence of log *k*_cat_/*K*_PRPP_.

The pH dependence of the kinetic parameters was evaluated to probe acid-base catalysis in the *Mt*UPRT mode of action. The pH-rate profile for *k*_cat_ was best fitted to an equation for bell-shaped curve: log*y* = log[*C*/(1+*H*/*K*_a_+*K*_b_/*H*)], where *y* is the kinetic parameter (*k*_cat_), *C* is the pH independent value of *y*, *H* is the proton concentration, and *K*_a_ and *K*_b_ are, respectively, the apparent acid and base dissociation constants for the ionizing groups. The bell-shaped pH profile for *k*_cat_ indicates participation of a single ionizing group in the acidic limb (slope value of +1) that must be unprotonated for catalysis, and participation of a single ionizing group for the basic limb (slope value of −1) that must be protonated for catalysis. Data fitting yielded p*K* values of 5.7 (±0.5) and 8.1 (±0.8). This result indicates that probably Asp198 and Arg102 of *Mt*UPRT (Fig. 6) are involved in catalysis (Fig. 11A). A catalytic mechanism has been proposed for UPRT from *B. caldolyticus* in which the O_2_ of the tautomeric enol form of uracil donates a hydrogen forming a hydrogen bond with the carboxylate group of aspartate (Asp200 for *B. caldolyticus* UPRT) and the α-phosphate group of PRPP, thereby simultaneously activating uracil as a nucleophile and PP_i_ as a leaving group [16]. It is just tempting to suggest that Asp198 in *M. tuberculosis* UPRT plays the role of Asp200 in *B. caldolyticus* UPRT. The role played by Arg102 of *Mt*UPRT in catalysis will have to await site-directed mutagenesis to provide solid experimental data.

The *k*_cat_/*K*_M_ data for PRPP (Fig. 11B) were fitted to the following equation: log*y* = log[*C*/(1+*K*_b_/*H*)]. This equation describes pH-rate profiles that show a decrease in log*y* with a slope of −1 as the pH values increase, in which *y* is the apparent kinetic parameter, *C* is the pH-independent plateau value of *y*, *H* is the hydrogen ion concentration, and *K_b_* is the apparent base dissociation constant for ionizing groups. Data fitting of pH dependence of log *k*_cat_/*K*_M_ for PRPP to this equation yielded a single ionizing group with a p*K* value of 9.5 (±1.1) that must be protonated for substrate binding (Fig. 11B). This result indicates that either Arg77 or Arg102 of *Mt*UPRT may play a role in PRPP binding (Fig. 6). These residues were previously shown to be conserved among UPRTs from different organisms, such as *T. gondii*, *B caldolyticus* and *E. coli* [46]. Although there is a high conservation of residues involved in ligand binding and catalysis (Fig. 6), amino acid sequences of different UPRT species are fairly dissimilar with identities ranging from 20 to 45% [31], [47].

The dependence of *k*_cat_/*K*_M_ for uracil on different pHs could not be analyzed because the saturation curves for uracil at pH values ranging from 7.0 to 8.5 fitted to a sigmoidal curve. Since the enzyme-catalyzed chemical reaction at these pH values does not obey Michaelis-Menten kinetics, it was not possible to determined *K*_M_ values.

## Materials and Methods

### Amplification and cloning of the *M. tuberculosis upp* gene

Two oligonucleotides (5′-ACCATATGCAGGTCCATGTCGTTGACCA-3′ and 5′-GTGGATCCTCAGCGCGGGCCGAACTG-3′) complementary to the amino-terminal coding and carboxy-terminal noncoding strands of *M. tuberculosis upp* gene were designed to, respectively, contain *Nde*I and *Bam*HI restriction sites (underlined). These primers were used to PCR amplify the *upp* gene from *M. tuberculosis* H37Rv genomic DNA. The PCR product, in agreement with the expected size (624 bp), was cloned into the pCR-Blunt cloning vector (Invitrogen) and subcloned into the pET-23a(+) expression vector (Novagen). The recombinant plasmid (pET-23a(+)::*upp*) was analyzed by automatic DNA sequencing.

### Expression and purification of recombinant *Mt*UPRT

The pET-23a(+)::*upp* recombinant plasmid was transformed into BL21(DE3) *E. coli* electrocompetent (Novagen) host cells and selected on LB agar plates containing 50 µg mL^−1^ ampicillin. A single colony was used to inoculate 50 mL LB medium containing 50 µg mL^−1^ ampicillin and grown overnight at 37°C. This liquid culture was used to inoculate 500 mL of LB medium (in a 2 L flask) containing 50 µg mL^−1^ ampicillin and grown at 37°C and 180 rpm up to an OD_600 nm_ of 0.4. Cells were grown for an additional period of eighteen hours (with no IPTG induction), harvested by centrifugation at 15,900 *g* for 30 min at 4°C, and stored at −20°C. The same protocol was employed for BL21(DE3) *E. coli* electrocompetent host cells transformed with empty pET-23a(+) expression vector, as control. The expression of *Mt*UPRT was analyzed by 12% SDS-PAGE stained with Coomassie Brilliant Blue [50].

The cell pellet (2 g of wet cells) was suspended in 20 mL of 50 mM Tris pH 7.6 (buffer A) containing lysozyme (0.2 mg mL^−1^) and incubated for 30 min at 4°C. Cells were disrupted by sonication and cell debris was removed by centrifugation (48,000 *g* 30 min 4°C). The supernatant was treated with 1% (wt/vol) streptomycin sulfate, stirred for 30 min, and centrifuged (48,000 *g* 30 min 4°C). The resulting supernatant, containing soluble *Mt*UPRT, was dialyzed against buffer A. An FPLC Äkta Purifier system (GE Healthcare) was utilized in all purification steps at 4°C. The dialyzed crude extract was loaded on a DEAE Sepharose CL6B anion exchange column (GE Healthcare) previously equilibrated with buffer A and the adsorbed material eluted with a linear gradient from 0 to 350 mM NaCl in buffer A at a 1 mL min^−1^ flow rate. Fractions containing the target protein were pooled (157.5 mL), concentrated (8.0 mL) using an Amicon ultrafiltration membrane (10,000 Da molecular weight cut off) (Millipore), and loaded on a HiPrep 26/60 Sephacryl S-300 gel filtration column (GE Healthcare). The target protein was isocratically eluted with buffer A at 0.25 mL min^−1^ flow rate. Pooled fractions (26 mL) were loaded on a Mono Q 16/10 anion exchange column (GE Healthcare) and protein elution was achieved with a linear gradient from 0 to 350 mM NaCl in buffer A. The pooled sample was dialyzed against buffer A and concentrated using an Amicon ultrafiltration membrane (10,000 Da molecular weight cut off). Homogeneous recombinant *Mt*UPRT protein was immediately frozen in liquid nitrogen and stored at −80°C. All protein purification steps were analyzed by 12% SDS-PAGE stained with Coomassie Brilliant Blue [48] and protein concentration was determined by the method of Bradford using the Bio-Rad protein assay kit and bovine serum albumin as standard (Bio-Rad Laboratories) [49].

### Mass spectrometry analysis and N-terminal amino acid sequencing

The subunit molecular mass of homogeneous recombinant *Mt*UPRT protein was assessed by mass spectrometry, using a MALDI-TOF/TOF on an ABI 4700 Proteomics Analyzer, an Ultraflex II (Bruker Daltonics), and a Q-TOF Ultima API (Micromass) as described elsewhere [50]. The N-terminal amino acid residues of homogeneous *Mt*UPRT were identified by automated Edman degradation sequencing using a PPSQ 23 protein peptide sequencer (Shimadzu).

### Determination of *Mt*UPRT molecular mass

#### Analytical ultracentrifugation

Analytical ultracentrifugation (AUC) experiments were performed with a Beckman Optima XL-A analytical ultracentrifuge using an AN-60Ti rotor at 20°C and analyzed as described elsewhere [51]. Experiments were carried out from 3,000 to 11,000 rpm at 4°C with scan data acquisition at 275 nm and protein concentration from 500 to 1,500 µg mL^−1^ in 100 mM Hepes pH 7.5 containing 10 mM MgCl_2_ and 150 mM NaCl. Sedimentation equilibrium (SE) analysis involved fitting a model of absorbance versus cell radius data by nonlinear regression using the Origin software package. The self-association method was used to analyze the experiments with several models of association for UPRT. The distribution of the protein along the cell was fitted to the following equation: *C* = *C*_o_exp[*M*(1−*V*_bar_ρ)ω^2^(*r* ^2^–*r*_o_)/2*RT*], in which *C* is the protein concentration at radial position *r*, *C*_o_ is the protein concentration at radial position *r*_o_, *M* is the molecular mass, *V*_bar_ is the protein partial specific volume, ρ is the buffer density, ω is the centrifugal angular velocity, *R* is the gas constant, and *T* is the absolute temperature. The Sednterp software was used to estimate protein partial specific volume and buffer density at 4°C.

#### Size exclusion chromatography

The molecular mass of native *Mt*UPRT was estimated by size exclusion chromatography on a Superdex 200 HR column (1.0 cm×30 cm) (Amersham Biosciences). The column was calibrated with the following protein standards (Amersham Biosciences): ribonuclease A (13,700 Da), carbonic anhydrase (29,000 Da), ovalbumin (43,000 Da), conalbumin (75,000 Da), aldolase (158,000 Da), ferritin (440,000 Da) and thyroglobulin (669,000 Da). Proteins were eluted from the column with 100 mM Hepes pH 7.5 containing 10 mM MgCl_2_, at a flow rate of 0.4 mL min^−1^ and the eluate was monitored at 215 and 280 nm. Blue Dextran was used to determine the void volume (*V*_0_). The *K*_av_ value was calculated for each protein using the equation (*V*_e_ – *V*_0_)/(*V*_t_ – *V*_0_), where is *V*_e_ the elution volume for the protein and *V*_t_ is the total bed volume, and *K*_av_ was plotted against the logarithm of standard molecular weights.

### Evaluation of pyrimidine bases as substrates using a discontinuous assay

Reaction mixtures containing 50 mM Tris pH 7.8, 10 mM MgCl_2_, 1 mM PRPP, and 0.1 mM of the pyrimidine base to be tested (uracil, cytosine, or thymine) were initiated by the addition of 54 nM of homogeneous *Mt*UPRT. The reactions were incubated at 37°C for 30 min and then boiled for 3 min to stop the reaction. The mixtures were passed through a Centricon (10,000 Da molecular weight cut off) to remove the protein content prior to analysis. The nucleotide contents of the samples were analyzed using an HPLC Äkta Purifier system and a Sephasil peptide C18 5 µm ST 4.6/250 column. A 500 µL aliquot of each sample was loaded on the column and adsorbed material isocratically eluted with 5 mM potassium phosphate pH 4.0 containing 5% acetonitrile for 10 min at 1 mL min^−1^flow rate. Nucleotides and bases were monitored at 254, 260, and 280 nm.

Another method used to analyze the nucleotide content was the LC-ESI-MS/MS. This experiment was employed to confirm results obtained in the analysis described above. The chromatography was carried out with an Eclipse plus C18 4.6/150 column (Agilent). The injected sample volume was 20 µL, which was eluted isocratically with 10 mM ammonium acetate containing 40% acetonitrile at 0.8 ml min^−1^ flow rate. The LC detector was an ESI coupled to the 3200 Q-Trap (Applied Biosystems MDS SCIEX), employing the ESI-MS/MS parameters as described by others [38]. During the chromatography run, precursor ion scan (Prec) and enhance product ion scan (EPI) were monitored. Prec monitored precursors of mass over charge ratio (*m/z*) of compounds containing a phosphate group (H_2_PO_4_^−^, *m/z* 97) and EPI gave the fragmentation spectra of the nucleotides *m/z* [38].

### Initial velocity measurements of recombinant *Mt*UPRT by a continuous assay

*Mt*UPRT enzyme activity was determined spectrophotometrically by measuring the conversion of uracil into UMP essentially as described by others [52] with a few changes. Enzyme activity measurements were performed using a UV-2550 UV/Vis Spectrophotometer (Shimadzu) at 25°C, and reactions initiated by the addition of enzyme to assay mixtures containing 10 µM uracil, 100 µM PRPP, 5 mM MgCl_2_, 100 mM Hepes pH 7.5, and 10 mM DTT in a final volume of 0.5 mL, and time courses followed for 60 s. This assay was based on the differential molar absorption between uracil and UMP at 280 nm (Δε = 2.5×10^3^ M^−1^ cm^−1^), in which an increase in absorbance is observed due to the formation of UMP. One unit of *Mt*UPRT is defined as the amount of enzyme that catalyses the conversion of 1 µmol of uracil in UMP per min.

### Evaluation of nucleotides as allosteric effectors

*Mt*UPRT activation or inhibition by allosteric effectors was evaluated and the following nucleotides were tested: 500 µM GTP, 500 µM CTP, 500 µM ATP, 500 µM UTP, and 100 µM UMP (Sigma-Aldrich). The experimental conditions were 35 µM uracil, 350 µM PRPP, 5 mM MgCl_2_, 10 mM DTT, 100 mM Hepes pH 7.5, and 112 nM *Mt*UPRT, using 0.5 cm pathlength quartz cuvettes. Enzyme activity was measured for 128 s and data collected every 5.6 s as described for the standard reaction.

### Kinetic parameters and initial velocity pattern

Determination of the steady-state kinetic parameters, *k*_cat_ and *K*_M_, was carried out at varying concentrations of one substrate while the concentration of the other substrate was fixed at constant saturating level. The concentrations of uracil were 2, 3, 6, 8, 10, 12 ,and 20 µM at a fixed PRPP concentration of 100 µM, while the concentrations of PRPP were 6, 20, 40, 60, 80, and 100 µM at a fixed uracil concentration of 10 µM. The reaction was initiated by adding 108 nM *Mt*UPRT and monitoring the change in absorbance at 280 nm for 60 s. Steady-state kinetic parameters were also determined in the presence of 100 µM GTP. Initial velocity patterns were also determined from measurements of *Mt*UPRT activity in the presence of varying concentrations of PRPP (6–100 µM) at several fixed-varied concentrations of uracil (2–10 µM).

### Isothermal titration calorimetry (ITC)

ITC experiments were carried out using an iTC_200_ Microcalorimeter (MicroCal Inc). Ligands and enzyme were prepared in 100 mM Hepes pH 7.5 containing 10 mM MgCl_2_. For direct titrations the sample cell was filled with 139 µM of *Mt*UPRT (200 µL) and titrated (39.7 µL) with different concentrations of either substrates or products: 200 µM of uracil, 500 µM of PRPP, 500 µM of UMP, and 350 µM of PP_i_. In addition, titration was performed with 10 mM of GTP and the sample cell was filled with either free *Mt*UPRT (139 µM) or *Mt*UPRT (139 µM) incubated with 100 µM PRPP for 1 hour before starting the measurements. Reverse titrations were also carried out where the sample cell was filled with either 90 µM of PRPP or 150 µM of UMP and titrated with 633 µM of UPRT.

A stirring speed of 500 rpm and a temperature of 25°C were employed for all ITC experiments. For direct titrations, the first injection (0.5 µL) was not used in data analysis and it was followed by either 17 injections (2.2 µL) for uracil, PRPP, PP_i_, and GTP or 21 injections (1.85 µL) for UMP. For the reverse titrations, the first injection (0.5 µL) was not used in data analysis and it was followed by either 30 injections (1.3 µL) for PRPP or 24 injections (1.6 µL) for UMP. The corresponding heat of dilution of each ligand (direct titrations) or UPRT (reverse titrations) titrated into buffer was used to correct data. The experimental data were evaluated using the Origin 7 SR4 software (MicroCal).

### pH-rate profiles

The pH dependence of the kinetic parameters was determined by measuring initial velocities in the presence of varying concentrations of one substrate and a saturating level of the other, in a buffer mixture of Mes/Hepes/Ches over the following pH values: 6.0, 6.5, 7.0, 7.5, 8.0, 8.5, and 9.0 [53]. Prior to performing the pH-rate profile determinations, the enzyme was incubated over this pH range and assayed under standard conditions to identify denaturing pH values and to ensure enzyme stability at the tested pH range. The pH-rate data were plotted as the dependence of either log*k*_cat_ or log*k*_cat_/*K*_M_ on pH values.

## Conclusions

Efficient prophylactic strategies are urgently needed to decrease the global incidence of TB. Live attenuated strains to be used as a vaccine offer a great promise against intracellular pathogens [54]. An ideal vaccine candidate would lead to limited replication *in vivo*, have the potential to induce immune response and improved safety comparing to BCG vaccine [55]. *Mt*UPRT is a key enzyme of the pyrimidine salvage pathway that might be an attractive target for the development of attenuated strains. Accordingly, the biochemical studies on *Mt*UPRT mode of action here described provide a solid support on which to base future efforts on gene replacement towards the development of efficient prophylactic strategies to combat TB. Moreover, attempts to ascertain the role of *Mt*UPRT in *M. tuberculosis* survival *in vivo* during latent TB is also worth pursuing. Understanding the mode of action of *Mt*UPRT may also be useful to chemical biologists interested in designing function-based chemical compounds to elucidate the biological role of this enzyme in the context of whole *M. tuberculosis* cells.
